# Detection and Impact of Rare Regulatory Variants in Human Disease

**DOI:** 10.3389/fgene.2013.00067

**Published:** 2013-05-31

**Authors:** Xin Li, Stephen B. Montgomery

**Affiliations:** ^1^Department of Pathology, Stanford University School of MedicineStanford, CA, USA; ^2^Department of Genetics, Stanford University School of MedicineStanford, CA, USA

**Keywords:** rare variant, genetics of gene expression, RNA-sequencing, Mendelian disorders, eQTLs, allele-specific expression

## Abstract

Advances in genome sequencing are providing unprecedented resolution of rare and private variants. However, methods which assess the effect of these variants have relied predominantly on information within coding sequences. Assessing their impact in non-coding sequences remains a significant contemporary challenge. In this review, we highlight the role of regulatory variation as causative agents and modifiers of monogenic disorders. We further discuss how advances in functional genomics are now providing new opportunity to assess the impact of rare non-coding variants and their role in disease.

## Introduction

In the next few years, hundreds of thousands of genomes will be sequenced, exposing an unprecedented wealth of genetic information. As each new genetic variant offers a potential window into an individual’s past, present, and future by providing insight into ancestry, traits, and disease risk, a major challenge will be to connect genetic variants to their functional consequences. Essential to addressing this challenge are high-throughput assays which connect genetic variants to molecular and cellular phenotypes. For instance, by assessing the association of genetic variants with the expression level of nearby genes (Box 1), a broad spectrum of causal variants, while potentially still unobserved, can be localized and their functional impact ascertained. At the moment, this experimental setting provides us with the most comprehensive system to identify regulatory variants and model the functional spectrum of human variation. Furthermore, genome-wide association studies (GWAS) have increasingly utilized such information to connect disease-predisposing variants to genes (Charlesworth et al., 2009; Montgomery and Dermitzakis, 2011). The basic principle behind such analyses has been to identify if a disease-predisposing variant is also associated to the expression level of a nearby gene – thereby providing a mechanistic hypothesis for disease etiology. In this area, multiple methods have been developed to assess the relationship between genetic effects on gene expression and disease by assessing the sharing of association (Zhu et al., 2007; Emilsson et al., 2008; Schadt et al., 2008; Nica et al., 2010). However, methods based on association do not well address the functional impact of rare, private, and *de novo* variants that dominate the site frequency spectrum in human populations. The extent of which was highlighted by two recent exome-sequencing that uncovered as much as 95% of protein-coding variation is rare (MAF ≤ 5%) and among these rare variants are the majority of sites predicted to be deleterious (Nelson et al., 2012; Tennessen et al., 2012). However, both studies focused on protein-coding variants where the genetic code greatly facilitates prediction of functional impact and not in the non-coding regions of the genome where considerable trait-predisposing variation is expected to reside (Hindorff et al., 2009). A considerable future challenge will be to determine the functional impact of a deluge of rare non-coding variants – as either causative agents or modifiers of traits. In this review, we first discuss the impact of non-coding variants as causal and modifying agents of monogenic disorders and then describe how advances in functional genomics can facilitate discovery and interpretation of rare, private, and *de novo* variants underlying a complete spectrum of human traits and diseases.

Box 1 **Statistical techniques for detecting genetic effects on gene expression.**

Expression quantitative trait loci (eQTL) mapping requires three important steps:

**Data normalization:**

Normalization is critical to mitigating the influence of technical artifacts or “batch effects” (Johnson et al., 2007; Williams et al., 2007) which can lead to spurious eQTL findings (Akey et al., 2007; Breitling et al., 2008). For microarray data, there are numerous normalization techniques to cope with batch effects (Irizarry et al., 2003; Johnson et al., 2007; Leek and Storey, 2007; Kang et al., 2008). For RNA-seq data, read counts can be transformed to RPKM (Reads Per Kilobase of transcript per Million mapped reads) (Mortazavi et al., 2008) or FPKM (Fragments Per Kilobase of transcript per Million mapped reads) (Trapnell et al., 2012) to account for differences in quantification due to transcript length and sequencing library depth. However, the utility of this approach is largely beneficial when comparing quantification between genes. As eQTL studies compare gene or transcript expression across samples, variability due to differences in transcript length can generally be ignored. However, in addition to library depth, the influence of highly expressed genes across samples can be profound – for instance, given equal numbers of reads sequenced, if a single gene (or small subset of genes) accounts for 10% of all reads in one sample and 5% of the reads in another, many other, unrelated genes may appear differentially expressed between these samples because reads not sequenced for one gene will yield extra reads sequenced for other genes. Such effects can be mitigated directly by inspection and regression or by using PCA-based normalization methods to correct out hidden factors (Pickrell et al., 2010). However, such normalization may be effective for dealing with global sequencing biases, individual genes can still be significantly influenced by read mapping errors (Marioni et al., 2008). Fundamental to addressing this issue are specific and sensitive read mapping algorithms for RNA-Seq data and an appropriate selection of an objective function for evaluating their performance in eQTL discovery. Here, number of eQTL discoveries is not sufficient as mappers which are highly influenced by variation may be enriched in false discoveries – advanced approaches account for alternative mappings and/or gain support from sequencing data across the expressed loci.

**Association and linkage analysis:**

In the presence of genetic marker data, different statistical tests are used to identify eQTL *within* populations and/or families (approaches for dealing with admixed or multi-population study designs are not covered in this review). Generally, the expression trait is assumed to be additive where each allele contributes equivalently. For population data, the most commonly used approach is a single-marker to single-trait *linear* or *non-parametric* regression; this analysis can be performed by statistical packages like R or statistical genetics tools such as PLINK (Purcell et al., 2007). For family data, linkage approaches based on inferring identity-by-descent (IBD) or linkage maps among family members are applied. Such a test can be conducted among all sib-pairs (Haseman and Elston, 1972) or all available family members (Amos, 1994). Many linkage analysis tools such as MERLIN (Abecasis et al., 2002) and SOLAR (Almasy and Blangero, 1998) can perform such analysis. For mixed designs, where data are composed of many nuclear families, transmission disequilibrium tests can be applied using tools like QTDT (Abecasis et al., 2000). However, each study design and associated statistical test has different advantages and limitations – association tests are powerful for detecting and localizing eQTL for common variants whereas linkage analyses cannot well localize variants but can identify intervals harboring rare eQTL.

Despite the availability of tools and simple statistical methods that support eQTL discovery, there remain important caveats in their utilization. For instance, considerable statistical power advantages can be achieved by restricting tests to variants nearby a gene (also termed *cis-*eQTL discovery). However, the size of the selected interval and the number of genes tested can be as influential as differences in sample size and number of variants tested. Complementary approaches have aimed to increase statistical power by integrating information from genes sharing regulatory pathways (Schadt et al., 2003). Here, the principal is that *bonafide* variant effects will propagate to downstream targets. For genome-wide (or *trans-*eQTL) analyses, many of the same limitations as present in GWA studies are equally applicable – common covariates such as ethnicity, relatedness, age and sex are also confounding factors in eQTL studies and should be appropriately accounted.

Though eQTL mapping is methodologically similar to GWA or family based linkage analysis, it can also be computationally more challenging when huge number of genes and variants need to be tested. Some newly developed computational tools are specifically optimized for fast eQTL mapping such as Matrix eQTL (Shabalin, 2012) and fastMap (Gatti et al., 2009).

**Multiple testing correction:**

Given the large numbers of tests and a typically small sample size, multiple testing correction is critical for eQTL mapping. Commonly used methods are Bonferroni correction, FDR/q-value (Hochberg and Benjamini, 1995; Storey and Tibshirani, 2003) and permutation-base *p*-value adjustment (Churchill and Doerge, 1994). A general practice of permutation is to generate a *p*-value distribution by randomly shuffling phenotype labels (typical for adjusting *cis* *p*-value) or by generating synthetic genotypes and phenotypes (typical for adjusting *trans* *p*-value). Using a permutation approach, a false-discovery rate can be estimated.

## Importance and Challenges in Identifying Non-Coding Variants

Advances in characterizing genome function have highlighted that, per base, non-coding sequence is at least as important for biochemical function as coding sequence (Birney et al., 2007; Ward and Kellis, 2012a). However, there is marked difference in the number of disease-predisposing variants that have been identified in the coding versus non-coding genome; for instance, the Human Gene Mutation database lists only a few percent of previously identified variants as being non-coding and regulatory (Stenson et al., 2009). This difference is attributable to how each class of variant is identified. For coding variants, the genetic code and data regarding protein structure and function facilitate predictions of causality. One can immediately identify if a variant changes an amino acid, creates a premature stop codon, alters hydrophobicity, disrupts a canonical splice junction, or impacts the structure of a protein domain. Furthermore, historically high costs of sequencing have made it reasonable to focus exclusively on variant detection in coding sequences where gene structure is known. In contrast, for regulatory variants the size of the target region to survey is poorly defined. For example, it is not clear whether to screen a single kilobase from the transcription start site or 100 kb or whether to also include intronic and downstream sequence of a gene or even whether to assume the effect is not *cis*-acting at all and is located elsewhere in the genome. Complicating matters further is that given our incomplete understanding of regulatory architecture, even if a segregating non-coding variant is found, it has generally been difficult without further molecular biology-based experimentation to differentiate it from other variants and demonstrate its impact (Montgomery, 2009).

## Rare Non-Coding Variants Underlying Disease

Despite the challenges of detecting causal regulatory variants, several studies have implicated them as the principal drivers of monogenic disorders. One of the earliest examples of a regulatory variant associated with a monogenic disorder was discovered in an individual affected with β-thalassemia (Poncz et al., 1982). The affected individual was identified to have an A–C transversion in a TATA-box element. Since this discovery, multiple regulatory variants associated with thalassemia have been described (Giardine et al., 2007). However, it is frequently the case that segregating non-coding variants lack differentiating genomic annotation and have required functional assays to support their effect. Such assays have generally included a combination of protein-expression, competition, electrophoretic mobility shift, and reporter gene assays. While such techniques are labor intensive, they have been critical to furthering the identification of non-coding variants underlying monogenic disorders. For instance, in Bernard–Soulier syndrome, an affected individual was identified with a G–C transversion within a predicted GATA-box motif (Ludlow et al., 1996). Subsequent functional assays supported the role of this variant by demonstrating *in vitro* an 84% reduction in promoter activity for the C allele and significant reduction in GATA-1 occupancy. Another similar example was described where a psoriasis-linked variant was identified through sequencing and predicted to influence a RUNX1 binding site. Subsequent functional assays confirmed to that the variant altered RUNX1 binding activity and reporter gene expression when RUNX1 and its coactivator CBFβ are present (Helms et al., 2003). We have further reported over 100 such variants where predicted regulatory polymorphisms have been assayed using a combination of electrophoretic mobility shift and reporter gene assays (Griffith et al., 2008). Despite this evidence however the *in vivo* activity and pathological cell types are rarely tested. Furthermore, the lack of availability of robust genome-wide assays for regulatory variation has been a challenge that is only more recently being addressed by advances in sequencing.

## Non-Coding Variants as Disease Modifiers

Genetic modifiers can dramatically alter the penetrance of pathogenic mutations or influence the expressivity of disease. Modifiers need not be rare when involved in rare diseases but here we focus on their effect in monogenic disorders where we know the impact of modifiers is considerably influential. In monogenic diseases such as CPS1 deficiency, pathogenic and non-coding variant interaction can manifest strikingly different morbidities and mortalities ranging from asymptomatic to perinatal death (Klaus et al., 2009). This phenomenon is further well characterized by the phenotypic diversity diseases such as sickle cell anemia and cystic fibrosis. For instance, in sickle cell anemia, affected individuals can be protected by a higher level of HbF expression which substitutes function of a defected HBB gene (Steinberg and Adewoye, 2006). HbF is a gene coding fetal hemoglobin which is expressed only in residual levels in adults after a developmental switch to the adult hemoglobin gene HBB, however, adult HbF expression level is highly variable and genetically determined (Thein and Menzel, 2009). In cystic fibrosis, a genome-wide association study within affected individuals identified *IFRD1* as modulating disease phenotype by influencing pathogen defense and inflammation. Beyond modifier gene effects as in both sickle cell anemia and cystic fibrosis, pathogenic mutations and their associated disease phenotypes have been demonstrated to be modulated by multiple different types of modifier effects including variable expression of the primary disease gene itself as in thalassemia (Marzo et al., 2010; Sankaran et al., 2010) and allelic modulation of expression of defected and normal copies of a gene as in erythropoietic protoporphyria (Gouya et al., 1996). Table 1 lists several well studied Mendelian diseases and an example of their modifier effects.

**Table 1:** **Mendelian disorders modified by gene expression**.

Mendelian disorder with wide phenotypic diversity	Primary disease gene	Example of genetic modifier	Modifier effect	Reference
Cystic fibrosis	CFTR	IFRD1	Regulation of neutrophil effector function	Gu et al. (2009)
Sickle cell anemia	HBB	HbF	Substitutes function of HBB	Steinberg and Adewoye (2006)
Thalassemia	HBA, HBB	Promoter variant	Changes levels of HBA, HBB expressions	Weatherall (2001)
Hemochromatosis	HFE	TFR2	Co-modulator of hepcidin	Camaschella (2005)
Familial Hypercholesterolemia	LDLR	TNFRSF1B	Reduces shedding of the TNFRSF1B receptor	Geurts et al. (2000)
Hereditary deafness	DFNB26	DFNM1	Suppress DFNB26	Riazuddin et al. (2000)
Retinitis pigmentosa	RPGR	IQCB1	Interaction with RPGR	Fahim et al. (2011)
Familial Mediterranean fever	MEFV	MICA	MICA behaves as a stress-inducible self-antigen	Touitou et al. (2001)
Asthma drug response	ADRB2	Promoter variant	Alters ADRB2 receptor expression	Drysdale et al. (2000)
Gaucher disease	GBA	SCARB2	Causes extracellular excretion of GCase	Velayati et al. (2011)
Adrenoleukodystrophy	ABCD1	SOD2	Modulates the response of neurons to oxidative damage	Brose et al. (2012)
Alpha 1-antitrypsin deficiency	A1AT	NOS3	Regulates vascular tone	DeMeo (2004)
Wilson disease	ATP7B	PRNP	Produces prion protein also involved in transporting copper	Weiss et al. (2010)
Hereditary pancreatitis	PRSS1	SPINK1	Serine protease inhibitor	Weiss et al. (2003)
Polycystic kidney disease	PKD1, PKD2	ACE	Increase angiotensin II levels	Devuyst (2003)
Erythropoietic protoporphyria	FECH	Intronic variant	Reduces FECH activity	Gouya et al. (2006)

More recently, genome-wide surveys of allelic expression have highlighted the abundance of interaction between protein-coding and non-coding variation. These studies have estimated that as high as 20% of non-synonymous variants and 30% of genes have allelic-specific expression (ASE) (Dimas et al., 2008; Ge et al., 2009; Lappalainen et al., 2011; Montgomery et al., 2011). Through RNA-sequencing, allelic expression of protein-coding variants can be routinely assessed and offers the potential to survey this type of interaction for rare or *de novo* variants without requiring variant phasing (Pastinen, 2010). When the correct pathological tissue is interrogated, it is expected that future studies which integrate allelic expression data for their pathogenic mutations will routinely have improved capacity for interrogating the interaction between protein-coding and non-coding variation and predicting disease expressivity.

## Assigning Function to Rare Non-Coding Variants

### Linkage analysis for gene expression

Linkage analysis is used in family based studies to detect *cis-* and *trans*-acting variants affecting genes expression. Gene expression level can be treated as a quantitative trait and analyzed by a general purpose linkage tool such as FBAT (Laird et al., 2000), GENEHUNTER TDT (Kruglyak et al., 1996), HRRR/HHRR (Terwilliger, 1995), LAMP (Li et al., 2005), MENDEL (Lange et al., 2005), PLINK (Purcell et al., 2007), PSEUDOMARKER (Hiekkalinna et al., 2011), QTDT (Abecasis et al., 2000), TRANSMIT (Clayton, 1999), UNPHASED (Dudbridge, 2008), and MERLIN (Abecasis et al., 2002). Many of these tools further model association in the presence of linkage and estimate the within family effect to the between family effects. Of these, the QTDT test has been well utilized in eQTL studies combining families and unrelated individuals (Cheung et al., 2010). A recent survey of the above methods has demonstrated improved power in the PSEUDOMARKER method relative to QTDT for which the authors account its ability to model true relationships between pedigrees, complete data usage and estimation of recombination, and allele frequencies from available data (Hiekkalinna et al., 2012). Using such methods, family studies have investigated the landscape of regulatory variants in different cell types such as lymphoblastoid cell lines (Schadt et al., 2003; Monks et al., 2004; Morley et al., 2004; Dixon et al., 2007; Cheung et al., 2010), lymphocytes (Göring et al., 2007), and adipose tissues (Emilsson et al., 2008). These studies have identified chromosomal regions co-segregating with transcriptome features, and most of them discover many eQTLs proximal to the genes they regulate. The reason why distal or *trans*-eQTLs are less detectable is in part due to a combination of smaller effect sizes and statistical power limitation by family size.

Despite restricted resolution in linkage analysis, family studies are especially useful for detecting potentially strong effect of rare variants. Deep sequencing of human exomes has detected an abundance of such rare variants (Nelson et al., 2012; Tennessen et al., 2012) in protein-coding sequences, however the extent to which rare variants have strong regulatory effects is only beginning to emerge. We recently reported increases in fold change for rare eQTLs when stratified by their derived allele frequency (Lappalainen et al., 2011). Existing linkage studies of gene expression are all based on families genotyped from SNP microarrays, yet to connect a discovered eQTL to a causal variant requires the use of deep sequencing data, which is also a necessary step for understanding the functional consequences of rarer variants.

### RNA-sequencing/ChIP-sequencing

**Figure 1:**
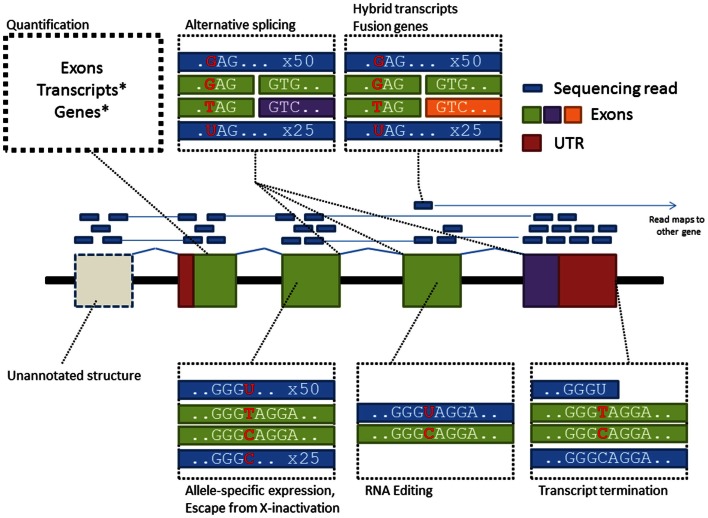
**RNA-Seq supports the characterization of diverse transcriptome features providing increased ability for linking trait-predisposing variation to their mode of impact**. Each box in this figure highlights a specific-type of biological data that can be assessed from RNA-Seq data.

Compared with microarrays, RNA-sequencing can potentially cover gene activity over the whole transcriptome while providing higher resolution of transcript complexity (Figure 1). Two studies utilizing this new technology have discovered hundreds of genetic effects on gene expression in European and African populations (Montgomery et al., 2010; Pickrell et al., 2010). Furthermore, utilizing RNA-sequencing reads spanning splice junctions, an indicator of alternative splicing events, studies have also shown wide spread splicing polymorphisms between individuals (Lalonde et al., 2011). However RNA-sequencing has its own technical biases (Mortazavi et al., 2008; Labaj et al., 2011), highly expressed genes may consume majority of reads which leaves many genes below quantifiable threshold, mapping artifacts may introduce false positive associations and technical biases like library construction and PCR-based amplification may further distort true allelic ratios. From the perspective of genome mapping, treatment of RNA reads is also substantially harder than DNA as reads from mRNA transcripts are gapped by introns; however, computational methods specifically designed for RNA-Seq data such as Tophat/Cufflinks tools (Trapnell et al., 2012) are increasingly available to support mapping and quantification of RNA reads for gene expression and alternative splicing analysis.

As gene expression is closely related to transcription factor binding, genetic studies of transcription factor binding offer insight into the functional of non-coding variants. Here, chromatin immunoprecipitation followed by high-throughput sequencing (ChIP-seq) can provide a comprehensive survey of transcription factor binding sites across the genome. ChIP-seq studies comparing related and unrelated individuals (McDaniell et al., 2010) have demonstrated heritability of chromatin structure and transcription factor binding, which has also been shown as a result of underlying genetic variation. Family information can be further utilized to distinguish the effects of rare variants and identify difference in binding of transcription factors specific to paternal and maternal alleles. One ChIP-seq study used phased diploid genomes from a family trio (Rozowsky et al., 2011) and by linking ASE to allele-specific binding of transcription factors connected expression of a gene to transcription factor binding on the same chromosome.

### Allele-specific expression

Allele-specific expression has also been shown to be a heritable trait under genetic control through family studies (Yan et al., 2002) and studies of monozygotic twins (Cheung et al., 2008), with 0.47–0.98 correlation between monozygote twins. Allelic expression is a more sensitive indicator of *cis*-acting effects (Pastinen et al., 2006; Ge et al., 2009) and can be used as supporting evidence for the presence of *cis*-regulatory variants near a gene (Montgomery et al., 2011). A specific advantage of the availability of functional genomic studies like RNA-Seq and ChIP-Seq using high-throughput sequencing has been that allelic effects can be ascertained *en masse* by assessing biases in sequencing reads over heterozygous positions (the null being that both alleles are equally present). However, robust calls of ASE typically require heterozygous sites to be identified through genotyping or DNA sequencing. Genotypes may be directly observed from RNA data, however due to systematic variation of read depth of RNA (since transcripts are expressed at different levels and have different sizes) and reference genome mapping biases (due to unobserved variants or homologous sequences) such approaches are error prone (Degner et al., 2009; Heap et al., 2010) and may further fail at sites which are monoallelically expressing and appear homozygous. Furthermore, as read depth is only evaluated at a single variant site, there can be considerable variation due to random sampling effect, which overshadows most allelic effects with small fold changes. We have previously used a modified binomial test to assess significance of skewed expression at a site (Montgomery et al., 2010). However, the power of the test to distinguish true ASE from random sampling is determined by read depth at that site. Due to variation in expression levels of different genes, only a small proportion of sites may reach required confidence level. Targeted approaches to assessing ASE have been reported which combining capture technology over previously identified heterozygous positions and may ultimately offer more uniform assessments of allelic effects (Zhang et al., 2009). The advantage of high-quality allelic expression information will provide extra information to aid in mapping *cis-*eQTL (Sun, 2012) and causal regulatory variants (Montgomery et al., 2011). For protein-coding variation implicated in disease, it will further demarcate the bounds of haploinsufficiency such that disease risk may be stratified by the levels of functional protein product an individual produces.

## Connecting Expression Quantitative Trail Loci to Human Disease

Most early attempts to connect expression to phenotypic traits in families have involved using standard Pearson correlation between the expression and trait measurements. However, despite the statistical simplicity of this methodology, it can inflate significance when there are few families and the trait and expression variance has a large between family component, and can reduce significance if there is a large within family component and no correlation between families. To address this, expression trait concordance tests which account for family structure have been reported (Kraft et al., 2003; Lu et al., 2004).

When combining genetic data to search for concordance of expression and trait association, an advantage is that the component of expression due to technical or environmental perturbation can be better controlled. We have previously reported methods that connect genetic effects on gene expression in unrelated samples to trait values by assessing if the genetic perturbation on expression is similar to the distribution of association scores for a trait (Nica et al., 2010). We have further developed a method to assess if there is an excess of causal regulatory variants of any frequency underlying a disease-associated variant (Conde et al., 2013). Here, our method tests if there are more ASE effects for heterozygotes of the disease-associated variant than homozygotes under the assumption that there may be one or more variants which stratify the risk and protective-associated alleles. Such approaches are now further complemented by the increasing amount of non-coding annotation from ChIP-Seq data and associated methods have been developed to determine the context and impact of trait-associated variants on epigenetic states (Boyle et al., 2012; Ward and Kellis, 2012b).

## Future Perspectives

As genome studies have been identifying large numbers of rare variants, it is expected that new methods and data will be required to uncover the impact of these variants and their involvement in diseases and traits. One of the most promising future technologies which will aid in interpreting the effect of rare regulatory variants in different tissues and developmental stages will be through the generation of induced pluripotent cells where gene expression can be assayed in multiple stages and differentiated cell types. Complementing this will be further advances in sequencing methods which provide phasing information – these advances will position rare and *de novo* mutations on the correct haplotype background and aid in investigations of genetic interaction. Furthermore, advances in ChIP-Seq and RNA-Seq will better aid in characterization of genetic effects on transcription factor binding and isoform expression and will ultimately unlock more complex functional interactions that underlie the etiology of diverse traits.

## Conflict of Interest Statement

The authors declare that the research was conducted in the absence of any commercial or financial relationships that could be construed as a potential conflict of interest.
